# *Staphylococcus aureus* lipoproteins in infectious diseases

**DOI:** 10.3389/fmicb.2022.1006765

**Published:** 2022-10-03

**Authors:** Majd Mohammad, Abukar Ali, Minh-Thu Nguyen, Friedrich Götz, Rille Pullerits, Tao Jin

**Affiliations:** ^1^Department of Rheumatology and Inflammation Research, Institute of Medicine, Sahlgrenska Academy, University of Gothenburg, Gothenburg, Sweden; ^2^Section of Medical and Geographical Infectiology, Institute of Medical Microbiology, University Hospital of Münster, Münster, Germany; ^3^Department of Microbial Genetics, Interfaculty Institute of Microbiology and Infection Medicine Tübingen (IMIT), University of Tübingen, Tübingen, Germany; ^4^Department of Clinical Immunology and Transfusion Medicine, Sahlgrenska University Hospital, Gothenburg, Sweden; ^5^Department of Rheumatology, Sahlgrenska University Hospital, Gothenburg, Sweden

**Keywords:** lipoproteins, lipopeptides, *Staphylococcus aureus*, infection, immunity, TLR2, host-pathogen interactions, metabolic fitness

## Abstract

Infections with the Gram-positive bacterial pathogen *Staphylococcus aureus* remain a major challenge for the healthcare system and demand new treatment options. The increasing antibiotic resistance of *S. aureus* poses additional challenges, consequently inflicting a huge strain in the society due to enormous healthcare costs. *S. aureus* expresses multiple molecules, including bacterial lipoproteins (Lpps), which play a role not only in immune response but also in disease pathogenesis. *S. aureus* Lpps, the predominant ligands of TLR2, are important for bacterial survival as they maintain the metabolic activity of the bacteria. Moreover, Lpps possess many diverse properties that are of vital importance for the bacteria. They also contribute to host cell invasion but so far their role in different staphylococcal infections has not been fully defined. In this review, we summarize the current knowledge about *S. aureus* Lpps and their distinct roles in various infectious disease animal models, such as septic arthritis, sepsis, and skin and soft tissue infections. The molecular and cellular response of the host to *S. aureus* Lpp exposure is also a primary focus.

## Introduction

The Gram-positive bacterium *Staphylococcus aureus* (*S. aureus*) is mostly known as being associated with dreaded antibiotic-resistant infections, and rightly so, *S. aureus* plays a much broader role in human diseases. On the one hand, *S. aureus* colonizes nearly half of the human population, permanently or intermittently, as a commensal bacterium (Wertheim et al., 2005). On the other hand, *S. aureus* is able to rapidly manifest its highly pathogenic traits as soon as it invades our body, and frequently causes severe clinical infections in humans, such as osteomyelitis, infective endocarditis, infectious arthritis, metastatic abscess formation and device-related infections (Edwards and Massey, 2011; Tong et al., 2015). It is also well known as the leading cause of bloodstream infections (Edwards and Massey, 2011). However, the molecular bases of *S. aureus* transition from commensal to pathogen remain elusive. Thus, gaining a greater understanding of its virulence mechanisms and interaction with the host is of vital importance in order to combat infectious diseases by *S. aureus*.

*Staphylococcus aureus* is a very resourceful pathogen (Lowy, 1998) as it possesses an immense arsenal of virulence factors, which enable the bacterium to thrive as an opportunist in humans. By biological function, virulence factors can be categorized as toxins, enzymes, immune evaders, as well as adhesins (Jin et al., 2021). By origin, they are divided to a capsular polysaccharide (O’Riordan and Lee, 2004), bacterial surface proteins (Foster et al., 2014; Jin et al., 2021), cell wall components (Lowy, 1998; Xia et al., 2010), and extracellular toxins (Xu and McCormick, 2012). Each virulence factor may have multiple biological functions. The perfect combination of those bacterial components helps staphylococci to adhere to host cells/tissues, resist engulfment of phagocytes, lyse the leukocytes, escape the immune killing, and finally cause the systemic and focal infections in different organs.

In the context of septic arthritis that is mainly caused by *S. aureus*, numerous virulence factors of *S. aureus* as well as various host factors targeted by the bacterium have been widely studied lately (Fei et al., 2011, 2022; Ali et al., 2015a,b,c; Mohammad et al., 2016, 2019, 2020; Na et al., 2016, 2020; Baranwal et al., 2017; Fatima et al., 2017; Jarneborn et al., 2020). These, among other important *S. aureus* virulence factors, have been extensively reviewed elsewhere (Mohammad, 2020; Jin et al., 2021).

Among the wide array of bacterial molecules that *S. aureus* exhibits are the lipoproteins (Lpps), which represent a major class of surface proteins in this opportunistic pathogen (Nguyen and Götz, 2016). Thus far, up to 70 Lpps have been detected in *S. aureus*, and the number of Lpps vary within various *S. aureus* genomes (Shahmirzadi et al., 2016). It is now widely believed that Lpps, peptidoglycan (PGN; Krause, 1975; Hrsak et al., 1979; Muller-Anstett et al., 2010; Volz et al., 2010; Schaffler et al., 2014) and bacterial excreted DNA/RNA (Miyake et al., 2018) are the main immune stimulators. By contrast, lipoteichoic acid (LTA), previously mistakenly considered an immunostimulant because of its contamination with Lpps (Hashimoto et al., 2006a,b; Zahringer et al., 2008), is not a TLR2 agonist. Unfortunately, in many review articles and textbooks it is still described as such.

It is well known that Lpp maturation is of critical importance for pathogenicity, inflammation, and immune signaling (Stoll et al., 2005; Nguyen et al., 2015; Nguyen and Götz, 2016). Lpps also play an essential role in the bacterial survival under infectious conditions due to their broad range of functions, including nutrient- and ion acquisition (Schmaler et al., 2009; Nguyen and Götz, 2016; Shahmirzadi et al., 2016; Nguyen et al., 2020). Lately, *S. aureus* Lpps have been shown to display important, but also differential roles in various inflammatory or infectious *in vivo* settings. Such host–pathogen interactions are the main focus of this review article.

## Bacterial lipoproteins

*Staphylococcus aureus* Lpps consist of a lipid and a protein moiety. The lipid part is covalently linked to a cysteine residue in the N-terminal region, enabling anchoring of Lpps to the outer leaflet of the bacterial cytoplasmic membrane (Nguyen and Götz, 2016). Furthermore, the triacylated fatty acid structure of the lipid moiety is incorporated into the membrane, while the protein portion protrudes toward the cell wall and beyond (Nguyen and Götz, 2016; Shahmirzadi et al., 2016). In contrast to those of *S. aureus* and other Gram-positive bacteria, Lpps of Gram-negative bacteria are also lipid-anchored to the inner leaflet of the outer membrane (Braun and Rehn, 1969).

The lipid portion of Lpps in *S. aureus* serves as a microbe-associated molecular pattern (MAMP) component and alerts the innate immune system through detection by pattern recognition receptors (PRRs), mainly TLR2, in host cells (Nguyen and Götz, 2016; Nguyen et al., 2017). Despite the fact that the lipid moiety is embedded in the membrane, a minor proportion of mature Lpps in *S. aureus* tend to be released from the membrane, enter the cell wall, and parts of the lipidated structures can be exposed on the cell surface (Stoll et al., 2005). The lipid modification is an absolute requirement for the activation of the host immune signaling, as Lpps lacking the lipid structure display no such stimulatory activity (Stoll et al., 2005; Nguyen and Götz, 2016). Thus, the lipid moiety functions as an important danger signal to the host (Nguyen and Götz, 2016; Nguyen et al., 2017). Consistently, Lpps and/or lipopeptides are the predominant ligands of TLR2 (Aliprantis et al., 1999; Brightbill et al., 1999; Hashimoto et al., 2006a).

*Staphylococcus aureus* mutant strains deficient in pre-Lpp lipidation (Δ*lgt* mutant) are less virulent than their parental strains due to reduced pathogenicity (Schmaler et al., 2009, 2010; Nguyen and Götz, 2016; Mohammad et al., 2020, 2021). The various outcomes of Lpp originating from *S. aureus* are summarized in Tables 1, 2, reviewed in (Mohammad, 2020).

**Table 1:** *Staphylococcus aureus* lipoproteins and their distinct role in various *in vitro* settings.

Cell types	Species/compound	Outcome	References
*Human cells*			
– MonoMac6, – Pulmonary epithelial cell line (A549), – Umbilical vein endothelial cells	*S. aureus* Δ*lgt*	Impaired production of IL-1, IL-6, and MCP-1	Stoll et al. (2005)
MonoMac6	*S. aureus* Δ*lgt*	Diminished levels of TNFα and IL-10	Stoll et al. (2005)
MonoMac6	*S. aureus* Δ*lpl*	Attenuated induction of TNF and IL-6	Nguyen et al. (2015)
THP-1	Heat-killed *S. aureus* Δ*lgt*	Lower production of TNF, IL-1β and IL-8	Kang et al. (2011)
– MonoMac6 – TLR2-transfected HEK293 cells	Purified *S. aureus* SitC	Induction of TNF and IL-6 expression	Muller et al. (2010)
Whole blood	*S. aureus* Δ*lgt*	Impaired proliferation	Bubeck Wardenburg et al. (2006)
Blood serum	*S. aureus* Δ*lgt*	No difference	Bubeck Wardenburg et al. (2006)
HeLa cells	*S. aureus* Δ*lpl*	Increased cell invasion frequency	Nguyen et al. (2016)
*Murine cells*			
Peritoneal macrophages	*S. aureus* Δ*lgt*	Impaired TLR2-MyD88-mediated cytokine production of IL-1, IL-6, IL-10 and TNF	Schmaler et al. (2009)
Peritoneal macrophages	Purified *S. aureus* SitC	TLR2-MyD88-mediated induction of TNF and IL-6 expression	Kurokawa et al. (2009)
Keratinocytes	Purified *S. aureus* SitC	Induction of TNF and IL-6 expression	Muller et al. (2010)
Peritoneal macrophages	– Purified *S. aureus* Lpp – Synthetic lipopeptides	TLR2-mediated induction of MIP-2, KC, and MCP-1 with a quick and dose-dependent release	Mohammad et al. (2019)
– Peritoneal macrophages – Splenocytes	– Purified *S. aureus* Lpp – Synthetic lipopeptides	TLR2-mediated induction of TNFα	Mohammad et al. (2019)
Peritoneal macrophages	– Purified *S. aureus* Lpp – Synthetic lipopeptides	TLR2-mediated induction of PAI-1, but not TF	Mohammad et al. (2021)
– Peritoneal macrophages – Splenocytes	*S. aureus* Δ*lgt* Extracellular vesicles	Impaired TLR2-mediated production of MIP-2, TNFα and IL-6	Kopparapu et al. (2021)
Whole blood	*S. aureus* Δ*lgt*	No difference	Mohammad et al. (2019)
Activated macrophages	*S. aureus* Δ*lgt*	Impaired proliferation	Bubeck Wardenburg et al. (2006)
Blood serum	*S. aureus* Δ*lgt*	Downregulated expression of IL-6 and KC, but not MCP-1	Mohammad et al. (2020)
Peritoneal macrophages	Purified *S. aureus* Lpp + GFP-expressing *S*. *aureus*	No impact on phagocytosis capacity	Mohammad et al. (2019)
Bone marrow-derived dendritic cells	*S. aureus* Δ*lgt*	Impaired TLR2-MyD88-mediated expression of B-cell activating factor	Im et al. (2020)
Bone marrow-derived dendritic cells	Synthetic lipopeptides	Induced TLR2-MyD88-mediated expression of B-cell activating factor	Im et al. (2020)
HeLa	Purified *S. aureus* Lpp – without lipid moiety	Extended G2 phase cycle	Nguyen et al. (2016)
HaCaT	Purified *S. aureus* Lpp – without lipid moiety	Increased host cell invasion *via* activation of Hsp90 receptor	Tribelli et al. (2020)
*Others*			
Bovine mammary epithelial cells	*S. aureus* Δ*lgt*	Impaired TLR2-mediated production of TNF, IL-6, and CXCL8	Liu et al. (2022)

Lpp, lipoproteins; Δ*lgt*, deletion mutant of preprolipoprotein diacylglyceryl transferase; Δ*lpl*, deletion mutant of lipoprotein-like lipoprotein genes; GFP, green fluorescent protein.

**Table 2:** *Staphylococcus aureus* lipoproteins and their distinct role in different *in vivo* animal models.

Site/organ – administration	Species/compound	Outcome	References
*Murine models*			
Knee – intra-articular	Purified *S. aureus* Lpp	– Bone destruction 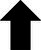	Mohammad et al. (2019)
Knee – intra-articular	*S. aureus* Δ*lgt*	– Knee swelling 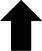 – Bacterial load 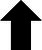	Mohammad et al. (2019)
Knee – intra-articular (co-injection)	Purified *S. aureus* Lpp + *S. aureus*	– Bone destruction 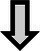 – Bacterial load 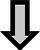	Mohammad et al. (2019)
Knee – intra-articular	– Purified *S. aureus* Lpp – Synthetic lipopeptides	– Bone resorption 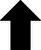	Schultz et al. (2022)
Femur – intraperitoneal	Synthetic lipopeptides	– Bone resorption 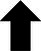	Kim et al. (2013)
Knee – intra-articular	*S. aureus* Δ*lgt* Extracellular vesicles	– Virulence 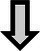	Kopparapu et al. (2021)
Septic arthritis – intravenous	*S. aureus* Δ*lgt*	– Virulence 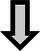 – Arthritis severity- no effect	Mohammad et al. (2020)
Sepsis – intravenous	*S. aureus* Δ*lgt*	– Virulence 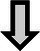	Schmaler et al. (2009)
Sepsis – intravenous	*S. aureus* Δ*lgt*	– Virulence 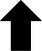	Bubeck Wardenburg et al. (2006)
Sepsis – intravenous	*S. aureus* Δ*lsp*	– Virulence 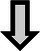	Bubeck Wardenburg et al. (2006)
Sepsis – intravenous	Synthetic lipopeptide pretreatment + methicillin-resistant *S. aureus* (MRSA)	– Bacterial load 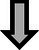 – Survival 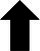	Huang et al. (2017)
Skin – subcutaneous	Purified *S. aureus* Lpp	– Skin inflammation 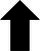	Mohammad et al. (2021)
Skin – subcutaneous	*S. aureus* Δ*lgt*	– Virulence 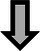	Mohammad et al. (2021)
Skin – epicutaneous	*S. aureus* Δ*lpl*	– Virulence 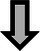	Nguyen et al. (2015)
Skin – intradermal	Purified *S. aureus* Lpp	– Virulence 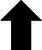	Saito and Quadery (2018)

Lpp, lipoproteins; Δ*lgt*, deletion mutant of preprolipoprotein diacylglyceryl transferase; Δ*lpl*, deletion mutant of lipoprotein-like lipoprotein genes; Δ*lsp*, deletion mutant of prolipoprotein signal peptidase.

### Biosynthetic pathway of *Staphylococcus aureus* Lpps

As an important part of the bacterial cell envelope homeostasis, lipidation of proteins naturally occurs as a posttranslational molecule reformation process, which ultimately leads to the formation of mature Lpps in both Gram-positive and Gram-negative bacteria (Buddelmeijer, 2015). Lpp modifications occur within the cytoplasmic membrane of the bacteria and involve the activity of the diacylglyceryl transferase (Lgt) and the signal peptidase (Lsp). In Gram-negative bacteria a third enzymatic step takes place that is catalyzed by the *N*-acyltransferase (Lnt), as described elsewhere (Nakayama et al., 2012; Buddelmeijer, 2015; Nguyen and Götz, 2016; Nguyen et al., 2020).

**Figure 1:**
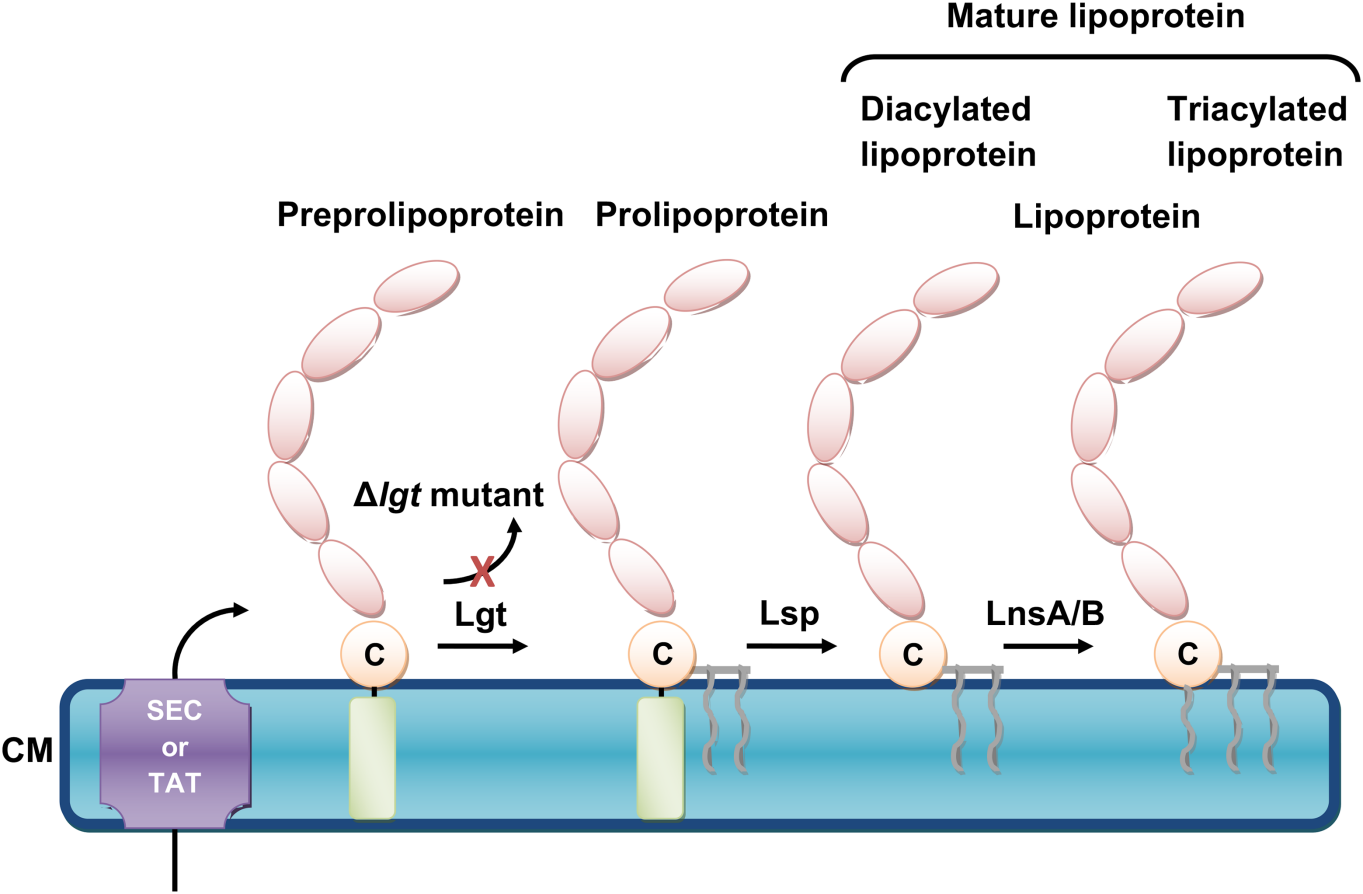
The biosynthetic pathway of *Staphylococcus aureus* lipoproteins. *S. aureus* lipoproteins are synthesized as preprolipoprotein precursors, which comprise an N-terminal signal peptide sequence (depicted as light-green cylinder), and are translocated across the cytoplasmic membrane (CM) by either the general secretory (Sec) or twin arginine translocation (TAT) pathways. The first enzyme, the preprolipoprotein diacylglyceryl transferase Lgt enables the transfer of a diacylglyceryl moiety to the indispensable cysteine residue (depicted as a beige circle with the letter, C), which forms a prolipoprotein. This lipid modification is followed by the second enzyme, the prolipoprotein signal peptidase Lsp., which cleaves the signal peptide and generates a mature diacylated lipoprotein. A third enzyme is required in order to form a mature triacylated lipoprotein. This lipid acylation is catalyzed by lipoprotein *N*-acylation transferase system LnsA/B. When *lgt* is deleted (Δ*lgt* mutant), the maturation of lipoproteins is inhibited and lipidation no longer occurs.

Lacking the *lnt* gene, *S. aureus* has been considered to produce only diacylated Lpps. However, with the development of gas chromatography–mass spectrometry (GC–MS) analysis, *S. aureus* was found to produce diacylated (Tawaratsumida et al., 2009) and triacylated Lpps (Kurokawa et al., 2009) depending on the environmental conditions (Kurokawa et al., 2012). Interestingly, in our previous studies, we identified SitC as triacylated Lpp in *S. aureus* (Nguyen et al., 2017) while Lpl1 from *S. aureus* SA113 was shown to exist both in a diacylated and triacylated form (Schultz et al., 2022). Recently, it was found that the *N*-acylation of Lpps in *S. aureus* and most likely many other Firmicutes, which lack *lnt*, is mediated by the two enzymes LnsA and LnsB (Gardiner et al., 2020). Figure 1 illustrates the Lpp biosynthesis pathway in *S. aureus*.

### The *Staphylococcus aureus* Lpp function

Lpps are characteristically divided into two functional entities, whereby the protein moiety serves and maintains the bacteria with its metabolic nutrition and function, whereas the lipid moiety has a key role in anchoring the protein into the bacterial membrane as well as in pathogenicity (Shahmirzadi et al., 2016; Nguyen et al., 2020).

The importance of *S. aureus* Lpps can be studied in numerous ways. Firstly, by inhibiting their maturation by mutating the specific catalytic enzymes, Lgt and Lsp. In the Δ*lgt* mutant the cysteine residue remains unmodified, hence preventing lipidation (Stoll et al., 2005). In the Δ*lsp* mutant, the first lipid modification of the Lpp is initiated; however, the signal peptide remains intact rather than cleaved (Nguyen and Götz, 2016). This ultimately leads to a disturbed balance within the Lpp biosynthetic machinery and may result in improper accumulation of immature Lpp.

**Figure 2:**
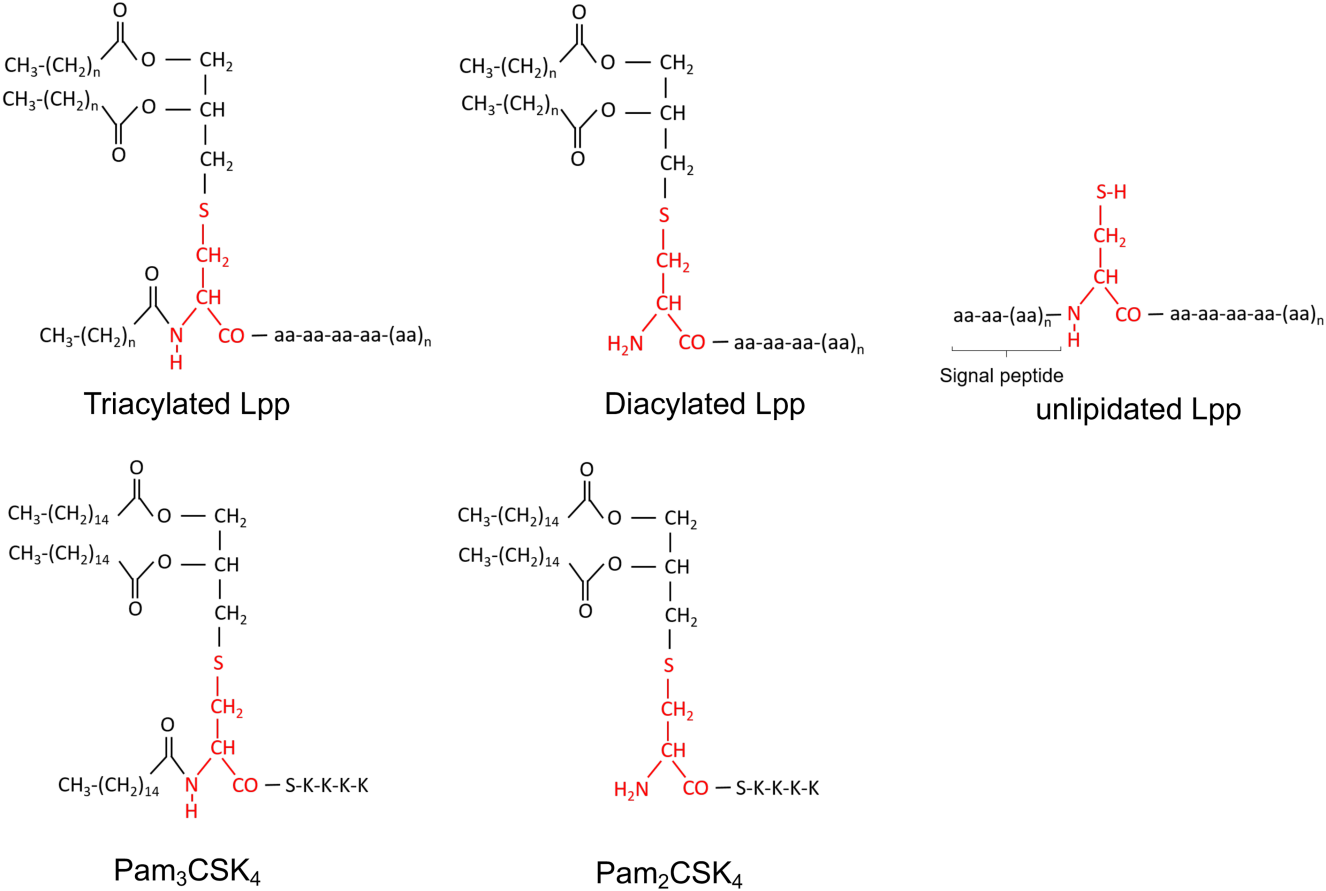
Chemical structure of *Staphylococcus aureus* Lpps (triacylated, diacylated and unlipidated lipoproteins) and synthetic peptides (Pam_2_CSK_4_ and Pam_3_CSK_4_). Cystein is marked in red.

Secondly, the significance of bacterial Lpps can also be investigated by isolation and purification of specific Lpps from the bacteria of interest, or thirdly, by using synthetic lipopeptides that resemble the lipid moiety structure of bacterial Lpp, such as Pam_3_CSK_4_ (triacylated lipid form) or Pam_2_CSK_4_ (diacylated lipid form). With regard to purified *S. aureus* Lpps, many pathogenic *S. aureus* strains harbor a genomic island, termed νSaα (Kuroda et al., 2001; Diep et al., 2006; Baba et al., 2008), that possesses highly conserved genes such as the lipoprotein-like cluster (*lpl*; Baba et al., 2008; Nguyen et al., 2015) encoding, among others, for the model lipoprotein Lpl1. The latter is denoted Lpl1(+sp) or Lpl1(−sp) depending on whether it carries or not the lipid moiety (Nguyen et al., 2015). Lately, Lpl1 has been extensively utilized in numerous studies to assess the importance of Lpps (Nguyen et al., 2015, 2016, 2017, 2018; Kumari et al., 2017; Mohammad et al., 2019, 2021; Tribelli et al., 2020; Kopparapu et al., 2021; Schultz et al., 2022). Figure 2 illustrate the structures and entities of Lpps as well as synthetic lipopeptides in *S. aureus*.

## The host immune response to Lpps in infections with *Staphylococcus aureus*

Despite its immense repertoire of virulence factors, upon host invasion, *S. aureus* alerts the host’s immune system and a battle between the host and the pathogen is immediately initiated. As mentioned, TLR2 serves as a critical receptor for Lpp and also recognizes synthetic lipopeptides (Aliprantis et al., 1999). Some of the innate and adaptive immune responses as well as complications that arise upon host recognition of *S. aureus* Lpps are briefly discussed below.

### *Staphylococcus aureus* Lpps and innate immunity

It is now well established that purified *S. aureus* Lpps trigger a rapid influx of innate immune cells, such as monocytes/macrophages and neutrophils, when injected in murine knee joints (Mohammad et al., 2019). A similar outcome with rapid recruitment of inflammatory cells was also observed in the skin model, as subcutaneous injection of *S. aureus* Lpps resulted in infiltration of neutrophils and monocytes/macrophages and induced skin lesions in mice (Mohammad et al., 2021). Intriguingly, leukocyte depletion by cyclophosphamide treatment was shown to diminish the Lpp-induced effect in the skin model. While PBS-treated control mice upon infection with *S. aureus* Newman parental strain, expressing Lpps, displayed more severe skin lesions and higher bacterial burden than mice infected with the derivative mutant strain Δ*lgt*, which lacks Lpp expression, the leukocyte depleted mice exhibited no such effect (Mohammad et al., 2021). This suggests that the effect induced by *S. aureus* Lpp expression in the skin model is dependent on the presence of leukocytes. Upon depletion of monocytes/macrophages in the purified *S. aureus* Lpp-induced local murine knee arthritis model, the arthritogenic and bone destructive effect was abolished, whereas neutrophil depletion played a minor role (Mohammad et al., 2019). This indicates that monocytes/macrophages are the main culprit behind the development of local knee arthritis in response to purified Lpps. Importantly, when purified Lpps and live *S. aureus* were co-injected into murine knee joints, bacterial eradication occurred. This was mediated by monocytes/macrophages and neutrophils, since depletions of these phagocytes resulted in aggravated disease severity and increased bacterial burden in local knee joints (Mohammad et al., 2019). Although *S. aureus* Lpps are known as potent stimulators of nitric oxide synthase and mediate nitric oxide production in mouse macrophages (Kim et al., 2015), our previous data showed that anti-nitric oxide synthase treatment had no impact on the bacterial clearance in knee joints (Mohammad et al., 2019).

TLR2 interacts differently with bacterial Lpps/lipopeptides depending on their lipid chain structure, although the downstream signaling events are conserved (Schenk et al., 2009). The N-terminal cysteine modification in bacterial Lpps (diacylated or triacylated structures) ultimately dictates the type of TLR2 hetero-complexes (with TLR1 or TLR6) that will form and lead to receptor activation (Gardiner et al., 2020). The elicited innate immune signaling, in turn, further modulates the host’s immune response and the inflammatory reactions (Nguyen and Götz, 2016). Importantly, the construction of *S. aureus* Δ*lgt* mutants along with improved methods for extraction and purification of Lpps (Stoll et al., 2005), have clearly proved that TLR2 is a major receptor exclusively activated by Lpps/lipopeptides (Bubeck Wardenburg et al., 2006; Hashimoto et al., 2006a,b). The TLR2 sensing ability varies considerably between different bacterial species (Nguyen and Götz, 2016). Nevertheless, among the immense range of virulence factors staphylococci dispose, Lpps are still considered as the main immunobiologically active components (Hashimoto et al., 2006a,b; Nguyen and Götz, 2016). It may seem paradoxical in bacterial evolution that Lpps evoke a cell-mediated immune response, specifically through TLR2-MyD88 signaling, thus initiating a battle with the host immune system. However, this skillful bacterium is known to utilize various strategies in order to escape immune recognition (de Jong et al., 2019; Cheung et al., 2021). A good example is the release of the staphylococcal superantigen-like protein 3 (SSL3), which is a TLR2 antagonist (Koymans et al., 2015). SSL3 prevents TLR2 dimerization with its co-receptors by forming a complex that partially closes off the pocket in TLR2, which consequently inhibits the binding between Lpps and TLR2 (Koymans et al., 2015).

### *Staphylococcus aureus* Lpps and adaptive immunity

In our previous work we showed that T-cells play a minor role in the severity of arthritis that follows intra-articular injection of *S. aureus* Lpl1 in the murine knee model, as CD4+ and CD8+ T-cell depletion as well as CTLA4-Ig treatment gave rise to similar outcomes in the treated groups and in the control group (Mohammad et al., 2019).

In *S. aureus-*induced skin infection model, a recent study showed that purified Lpps caused skin inflammation, accompanied by interferon γ producing T cell accumulation (Saito and Quadery, 2018). CD4+ T-cells have been suggested to offer protection against secondary *S. aureus* skin and soft tissue infections (Montgomery et al., 2014). However, it was recently demonstrated that skin tissues from mouse auricle upon challenge with purified *S. aureus* Lpps did not increase the migration levels of T-cells or NK cells in both C57BL/6 wild-type and TLR2 deficient mice (Mohammad et al., 2021).

In the case of Lpp-induced joint inflammation, intra-articular injection of *S. aureus* Lpps did not trigger the influx of either B- or T-cells (Mohammad et al., 2019). In *S. aureus* skin infection, B-cells are known to produce antibodies that are directed against *S. aureus* virulence factors, and thus mediate important immune responses against the pathogen (Krishna and Miller, 2012). However, in the context of the *S. aureus* Lpp-induced skin infection model, subcutaneous injection of the *S. aureus* Lpp-expressing strain induced the same effects with regard to the severity of the skin lesions and bacterial burden in mice deficient in B- and T-cells (SCID mice), as in Balb/c control mice (Mohammad et al., 2021). These findings indicate that the adaptive immunity plays a minor role in the Lpp-induced effects in murine skin infection.

## Lipoproteins and host immune response

### Importance of *Staphylococcus aureus* Lpps in iron acquisition and metabolic fitness

Lpp maturation is a necessity for many bacterial species in their battle against the host (Kovacs-Simon et al., 2011). Investigation of the pathogenic properties of Lpps has mostly been conducted through implementation of *lgt* or *lsp* mutants in various *in vitro* and *in vivo* settings. Important parameters, such as the ability to acquire ions and nutrients as well as bacterial survival and bacterial proliferation, have thus been studied to a large extent. Lpp maturation has been shown to be of fundamental importance in iron acquisition, stimulating *S. aureus* bacterial growth, as the *S. aureus* SA113 Δ*lgt* mutant strain exhibited growth defects compared to its parental strain under nutrient-restricted conditions (Stoll et al., 2005). In line with these results, we demonstrated that the Δ*lgt* mutant had impaired growth compared to its parental strain in nutrient-poor conditions, while the Δ*lgt* mutant and parental strain had similar growth rates in nutrient-rich conditions (Mohammad et al., 2020). Schmaler et al. demonstrated that upon repletion of iron sources, the SA113 Δ*lgt* mutant strain that had been grown in iron-depleted conditions exhibited impaired iron-dependent bacterial growth compared to its parental strain (Schmaler et al., 2009). Acquisition of iron is a pervasive feature employed by pathogens such as *S. aureus* to improve their survival and proliferation (Hammer and Skaar, 2011; Haley and Skaar, 2012). It is not surprising that among proposed 67 Lpp of *S. aureus* USA300, a highest number of Lpp are involved in iron transport (Shahmirzadi et al., 2016). It has been experimentally proved that *S. aureus* uses 5 transport systems to obtain iron from the environment including FhuCBG Fe chelator system with the involvement of 2 Lpp FhuD1 and FhuD2 (Sebulsky et al., 2000, 2004; Sebulsky and Heinrichs, 2001), SirABC iron regulator system with Lpp SirA (Heinrichs et al., 1999; Dale et al., 2004), IsdCDEF heme uptake system with Lpp IsdE (Mazmanian et al., 2002; Grigg et al., 2007), SstABCD siderophore transport system with Lpp SstD (Morrissey et al., 2000) and FepABC iron transport system with Lpp FepA (Biswas et al., 2009).

Overall, Lpps in *S. aureus* contribute to provide the bacteria with adequate amounts of iron under infectious conditions. As iron is an essential resource for *S. aureus* to survive and thrive, especially in the iron-depleted conditions of the infected human body (Hammer and Skaar, 2011; Sheldon and Heinrichs, 2012), inadequate maturation of Lpps can be detrimental for this pathogenic bacterium as the maintenance of the metabolic activity and fitness is threatened. The largest iron reservoir in the host is represented by the heme iron, which serves as the preferred iron source for *S. aureus* (Skaar et al., 2004). As *S. aureus* seeks ways to acquire sufficient iron uptake during an infection, the host innate immune system employs defensive mechanisms by limiting the iron availability to the invading bacteria, functioning as one of the primary host defense responses during infection (Haley and Skaar, 2012; Cassat and Skaar, 2013). *Staphylococcus aureus* can therefore utilize Lpps as an efficient iron transporter in order to circumvent this nutritional immunity, thus increasing its chances of survival and consequently of causing disease. The loss or disturbances of iron-regulated systems, including Lpp maturation, during *S. aureus* infections are strongly associated with attenuated virulence (Torres et al., 2006; Pishchany et al., 2014), which further implies the critical role iron serves in the pathogenesis of diseases this bacterium causes.

### *Staphylococcus aureus* Lpps – *in vitro* effects

The use of *S. aureus* Δ*lgt* mutants as well as purified Lpp compounds and synthetic lipopeptides have demonstrated the important role of Lpps in triggering the release of cytokines and chemokines as a response of the host immune system.

Synthetic lipopeptides are known to potently stimulate the secretion of various cytokines in human monocytes and macrophages (Hoffmann et al., 1988; Kreutz et al., 1997). In contrast to the parental strain, the *S. aureus* Δ*lgt* mutant displays reduced immune-stimulatory abilities in various human cells, such as the human monocytic cell line (MonoMac6), the human pulmonary epithelial cell line (A549), and in human umbilical vein endothelial cells, with consequent impaired production of pro-inflammatory cytokines and chemokines, including IL-6, IL-8, and monocyte chemoattractant protein 1 (MCP-1; Stoll et al., 2005). Furthermore, both TNFα and IL-10 levels were diminished over time in MonoMac6 cells (Stoll et al., 2005). In another human monocytic cell line, referred to as THP-1 cells, cell stimulation with heat-killed *S. aureus* Δ*lgt* mutant was associated with lower production of TNF, IL-1β and IL-8, as compared to those cells that were stimulated with the heat-killed *S. aureus* parental strain (Kang et al., 2011). Also, *S. aureus* Δ*lgt* mutants were associated with impaired TLR2-MyD88-mediated cytokine production (IL-1, IL-6, IL-10 and TNF) in mouse peritoneal macrophages, whereas the *S. aureus* parental strain induced early and strong cytokine release (Schmaler et al., 2009). Purified *S. aureus* SitC is well known to induce TNF and IL-6 expression in murine peritoneal macrophages in a TLR2-MyD88-dependent manner (Kurokawa et al., 2009). The release of such pro-inflammatory cytokines was also demonstrated in human monocytes and mouse keratinocytes, and in TLR2 expressing HEK cells (Muller et al., 2010).

We showed that stimulation of peritoneal macrophages with purified *S. aureus* Lpl1 induced a quick and dose-dependent release of the neutrophil chemoattractant, macrophage inflammatory protein-2 (MIP-2) and keratinocyte-derived chemokine (KC) as well as the monocyte chemoattractant, MCP-1 (Mohammad et al., 2019). These strong and rapid effects were observed already 4 h after stimulation and were dependent on the lipid- and not the protein moiety. In fact, purified Lpl1(+sp) induced similar levels as lipopolysaccharide, which served as the positive control, while Lpl1(−sp), lacking the lipid moiety, was only capable to exhibit similar stimulation levels as the negative medium control in terms of secretion of the assessed chemokines (Mohammad et al., 2019). These findings were only observed in the peritoneal macrophage supernatants collected from the C57BL/6 wild-type and not in the TLR2 deficient mice, and were thus mediated through the PRR TLR2 (Mohammad et al., 2019). The importance of *S. aureus* Lpp and its lipidation was further verified by the fact that the triacylated synthetic lipopeptide Pam_3_CSK_4_ induced similar MIP-2, KC and MCP-1 levels to the purified Lpl1(+sp) compound (Mohammad et al., 2019). In addition to the neutrophil- and monocyte chemoattractant chemokines, the pro-inflammatory cytokine, TNFα, was also induced in a TLR2-dependent manner upon Lpl1(+sp) and Pam_3_CSK_4_ stimulation of both mouse peritoneal macrophages and splenocyte cultures (Mohammad et al., 2019). Overall, our findings indicate that *S. aureus* Lpps are potent immune stimulators that induce rapid release of important chemokines and cytokines from immune cells exclusively *via* TLR2.

With respect to bacterial growth, our data revealed that SA113 parental strain and Δ*lgt* mutant strain proliferated similarly in mouse whole blood during a 2 h incubation (Mohammad et al., 2019), which are not in agreement with previous findings in human whole blood (Bubeck Wardenburg et al., 2006). However, parameters such as the assessed time points, different *S. aureus* strains (SA113 vs. Newman), and the different sources of whole blood (mouse vs. human) might explain these discrepancies. Nevertheless, in the model of systemic infection, the expression of mature *S. aureus* Lpp was responsible for a more systemic inflammatory status, whereby the Newman parental strain was associated with higher levels of IL-6 and KC, but not MCP-1 (Mohammad et al., 2020). No differences between the parental strain and the *lgt* deficient mutant strain were observed in TLR2 knockout mice, suggesting that the cytokine response was dependent on TLR2 (Mohammad et al., 2020). Although *S. aureus* Newman parental strain had higher bacterial proliferation in the presence of activated murine macrophages compared to the Newman Δ*lgt* mutant strain, the phagocytic capacity of macrophages was not affected by the Lpp expression (Bubeck Wardenburg et al., 2006). These results are in agreement with our data whereby purified *S. aureus* Lpl1 did not influence the phagocytic capacity of macrophages (Mohammad et al., 2019).

It was very recently demonstrated that *S. aureus* Lpps are the culprits behind the pro-inflammatory property of *S. aureus* extracellular vesicles (EVs) *in vitro*. Upregulated levels of MIP-2, TNFα and IL-6 were observed upon stimulation of murine peritoneal macrophages with Lpp-carrying EVs but not with Lpp-deficient ones, since EVs isolated from *S. aureus* Δ*lgt* mutant lacked the capacity to induce any immune stimulation (Kopparapu et al., 2021). A similar outcome was observed in an *in vitro* splenocyte stimulation setting (Kopparapu et al., 2021). As expected, this pro-inflammatory response to *S. aureus* Lpp EVs was shown to be TLR2-dependent (Kopparapu et al., 2021).

Gardiner et al. studied the importance of *lnsA/B* in the immune-stimulatory effects of *S. aureus* using HEK-TLR2 cells, and demonstrated that the expression of IL-8 increased ~10 times upon deletion of either of the two genes, *lnsA* or *lnsB* (Gardiner et al., 2020). This suggests that an intact *lnsA/B* system is advantageous for *S. aureus* to evade TLR2 immune recognition. Furthermore, various staphylococcal species possess varying lengths of the *N*-acylation at the N termini of the cysteine residue within the lipid moiety of Lpps: the opportunistic pathogens *S. aureus* and *S. epidermidis* carry a long-chain *N*-acylated fatty acid, while the non-commensal, non-pathogenic *S. carnosus* (Biswas et al., 2009; Rosenstein and Götz, 2013) carries a short-chain *N*-acylated fatty acid, which corresponds to a heptadecanoyl fatty acid and an acetyl fatty acid, respectively (Nguyen et al., 2017). Nguyen et al. revealed that *S. carnosus* was capable to induce 10-fold higher TLR2-mediated cytokine responses compared to *S. aureus* and *S. epidermidis* (Nguyen et al., 2017). Furthermore, both TNFα and IL-8 secretion were strongly upregulated by *S. carnosus* in MonoMac6 and HEK-TLR2 cells in comparison to several *S. aureus* strains, including SA113, HG003, and the MRSA strain, USA300 (Nguyen et al., 2017). This concept was further proved as the Lpp SitC that was purified from *S. carnosus* triggered higher TLR2-mediated induction of IL-8 than SitC that was extracted from *S. aureus* (Nguyen et al., 2017). This was also confirmed in human monocyte-derived dendritic cells whereby the levels of various pro-inflammatory cytokines were elevated in a similar manner (Nguyen et al., 2017). Overall, these findings are in line with previous reports that showed that different modifications of the lipid moiety trigger diverse TLR2 activations (Armbruster et al., 2019; Gardiner et al., 2020). Interestingly, the expression of other *S. aureus* components, more precisely capsular polysaccharide, can mask *S. aureus* Lpps and attenuate the recognition of Lpps and TLR2 activity (Hilmi et al., 2014).

It was recently demonstrated that Lpl alters the cell cycle in HeLa cells delaying the G2/M phase transition, which consequently leads to increased cell invasion (Nguyen et al., 2016). This effect was shown to be mediated by the secretion of cyclomodulin (Nguyen et al., 2016), which functions as bacterial toxin disturbing the regular course of the host cell cycle (Taieb et al., 2011). Cyclomodulin seems to be utilized by *S. aureus* not only to increase host invasion but also to induce bacterial proliferation within the host cells (Alekseeva et al., 2013). The factors that contribute to the effects of Lpl on host cells were recently addressed by Nguyen et al. (2018). In fact, it was revealed that not only the growth phase of *S. aureus* differentially affects the observed phenotypes but also that the host contributes to such effects since bacterial invasion frequency was higher in TLR2 expressing cells (Nguyen et al., 2018).

Nguyen et al. showed that deletion of the entire *lpl* operon in the *S. aureus* USA300 strain resulted in attenuated induction of pro-inflammatory cytokines in human monocytes, macrophages and keratinocytes, compared to the parental strain expressing the *lpl* gene cluster (Nguyen et al., 2015). To verify the observed phenomenon, the *lpl* cluster was cloned into another *S. aureus* strain, HG003, which naturally lacks *lpl* genes, and this resulted in increased production of the pro-inflammatory cytokines (Nguyen et al., 2015). The purified lipidated Lpp, Lpl1, was further shown to evoke a TLR2-dependent response (Nguyen et al., 2015). A recent study revealed that the cluster of *lpl* proteins in *S. aureus* was upregulated in a MRSA strain during sub-inhibitory exposure to β-lactam antibiotics (Shang et al., 2019). This suggests that *S. aureus* Lpps, more specifically, Lpl has a virulent nature resulting in enhanced pathogenicity of MRSA. *Staphylococcus aureus* Lpps and their differential roles in various *in vitro* settings are summarized in Table 1.

### *Staphylococcus aureus* Lpps – *in vivo* effects: The role in virulence and pathogenicity during host invasion

Besides their vital role in maintaining and upregulating the fitness of the bacteria (Shahmirzadi et al., 2016), bacterial Lpps possess a variety of key functions, some of which serve critical roles during infectious and inflammatory conditions. In most, but not all cases, maturation of Lpps has been strongly associated with enhanced pathogenic invasion, bacterial survival and immune activation (Nguyen and Götz, 2016; Shahmirzadi et al., 2016).

#### Intra-articular injection of *Staphylococcus aureus* Lpps causes joint inflammation

The importance of IL-1 in Lpl1-induced synovitis was recently assessed in mice through treatment with the IL-1 receptor antagonist (anakinra). It was shown that IL-1 did not play a major role in the induction of synovitis in a local arthritis model (Mohammad et al., 2019). The importance of TNF has also been studied in the context of *S. aureus* Lpp exposure. We explored whether TNF inhibition had any beneficial effects in the model of purified Lpp-induced synovitis by treating the mice with anti-TNF treatment (etanercept). TNF was indeed partially involved in modulating the arthritogenic effects in local *S. aureus* Lpl1-induced knee arthritis (Mohammad et al., 2019).

Decreased arthritis severity is closely associated with lower levels of IL-6 in local joints, suggesting that IL-6 is an important cytokine for maintenance of septic arthritis (Mohammad et al., 2019). We also demonstrated that *S. aureus* Lpps trigger the quick release of KC and MIP-2 in local tissues including knees and skin with enhanced influx of phagocytes, consequent inflammation and tissue damages (Mohammad et al., 2019, 2021).

We reported that *S. aureus* Lpps play various roles in different murine models. In a mouse model of *S. aureus* septic arthritis, Lpps gave rise to pronounced arthritogenic effects in both NMRI and C57BL/6 wild-type mouse strains, whereas TLR2 deficient mice displayed no signs at all following intra-articular knee joint challenge with the purified Lpl1(+sp) (Mohammad et al., 2019). The arthritogenic properties were thus mediated through TLR2 but also *via* the lipid portion of Lpp, since Lpl1(−sp), comprising only of the protein moiety, lacked the ability to cause any knee joint swelling. In addition to the observed long-lasting macroscopic arthritic effect in the Lpl1(+sp) group, which was detected already within 24 h after injection, Lpl1 contributed to local knee synovitis in a dose-dependent fashion. The histological sections revealed that Lpl1 induced synovitis even when administered at the nanogram level, indicating that *S. aureus* Lpps are highly potent. Intriguingly, when challenging the mice with an Lpl1 dose at the microgram level, all joints developed bone erosions within 10 days after injection (Mohammad et al., 2019).

Prior to this study, we reported that murine knee joints, challenged with antibiotic-killed *S. aureus*, displayed severe bone erosion and long-lasting arthritis (Ali et al., 2015c). This aspect is indeed very clinically relevant since patients suffering of septic arthritis are likely to develop irreversible permanent joint destruction, even after successful bacterial eradication through the standard treatment procedure (Goldenberg, 1998). Since the *S. aureus* cell wall components were responsible for causing this inflammatory effect in the joints, partially *via* TLR2 (Ali et al., 2015c), this prompted us to study the involvement of *S. aureus* Lpps in the host reaction. We were able to show that Lpl1 served as one of the inducers, and also elucidated the cellular mechanism by showing that monocytes/macrophages were the responsible cell type mediating the Lpl1-induced effect in the local knee joints through TLR2-dependent responses (Mohammad et al., 2019). This further strengthens the concept that the disease severity of septic arthritis is at least partially mediated by an exaggerated immune response that is triggered by specific bacterial components, such as Lpp (Mohammad et al., 2019), which is in agreement with previous reports (Deng et al., 1999; Ali et al., 2015c).

#### Intra-articular injection of *Staphylococcus aureus* Lpps causes focal bone resorption

Recently, we also demonstrated in a local knee arthritis model that *S. aureus* Lpp induced bone resorption in NMRI mice, an effect that was mediated through its lipid moiety and that was dependent on monocytes/macrophages (Schultz et al., 2022). Moreover, when challenging the mice with synthetic lipopeptides through intra-articular knee joint injection, the diacylated lipid moiety, Pam_2_CSK_4_, was more potent in inducing bone resorption than the triacylated lipid moiety, Pam_3_CSK_4_ (Schultz et al., 2022). In fact, we have recently showed that Lpl1(+sp), isolated from *S. aureus* SA113 strain, contains both diacyl and triacyl lipid-moieties (Schultz et al., 2022). A previous study demonstrated that intraperitoneal injection of synthetic lipopeptides, resembling the lipid structure of Lpps, were associated with severe bone loss in the femurs of mice (Kim et al., 2013). This further suggests that lipidated Lpps play a potent pathogenic role in the bone of mice independent of the route of administration.

Recently, we revealed that upon intra-articular knee joint injection of mice, Lpp-carrying EVs displayed pathogenic capacities by giving rise to more severe macroscopic arthritis as well as synovitis in two different mouse strains (NMRI and C57BL/6 wild-type mice), and that such effect was mediated by monocytes/macrophages *via* TLR2 (Kopparapu et al., 2021).

#### *Staphylococcus aureus* Lpps play distinct role in local- and hematogenous septic arthritis, and sepsis

In a local knee septic arthritis model triggered by intra-articular injection of living bacteria, the SA113 Δ*lgt* mutant strain, lacking Lpp expression, resulted in more pronounced knee joint swelling in comparison to its parental strain. In addition, this was accompanied by increased bacterial load as well as elevated IL-6 levels in the local infected knee joints (Mohammad et al., 2019). Conversely, in a mouse model of *S. aureus*-induced sepsis, inoculation with the same strain resulted in a lower bacterial burden in the knee joints of mice (Schmaler et al., 2009). Another study demonstrated that *S. aureus*, deficient in Lpp expression, causes bacterial immune evasion and lethal infections in a murine sepsis model (Bubeck Wardenburg et al., 2006). This clearly suggests once more that Lpps implement different behavior strategies in different animal models, depending on the transmission route, the assessed time points, and the examined organs. Indeed, in our well-established murine model of hematogenous septic arthritis, intravenous inoculation with the *S. aureus* Newman parental strain resulted in higher bacterial persistence in kidneys of both C57BL/6 wild-type and TLR2 deficient mice (Mohammad et al., 2020). In fact, this corroborates previous reports whereby more pronounced bacterial burden in different organs, including kidneys, was demonstrated in mice after infection with SA113 parental strain compared to the Lpp-deficient Δ*lgt* mutant strain, independent of TLR2 and MyD88 signaling, in a sepsis model (Schmaler et al., 2009). Moreover, a previous study demonstrated that increased bacterial persistence occurred in kidneys of Balb/c mice when infected with the USA300 MRSA parental strain in comparison to its Δ*lpl* mutant strain, lacking the *lpl* gene cluster (Nguyen et al., 2015). Furthermore, in a murine sepsis model, it was recently demonstrated that mice pre-treated with the synthetic lipopeptide Pam_3_CSK_4_ had decreased bacterial burden and increased survival following infection with a *S. aureus* MRSA strain (Huang et al., 2017).

#### The role of *Staphylococcus aureus* Lpps in skin infections

Defects in the skin barrier, due to breaches or abrasions in the skin tissue, enable *S. aureus* to penetrate into the damaged site and enter the underlying tissue, thus establishing a skin infection. It consequently proliferates on site, and release different bacterial components and toxins, causing symptoms. Directly after infection, leukocytes are rapidly recruited, and antimicrobial peptides are upregulated in the site of infection. Invasion into the host cells is one of the effective strategies for *S. aureus* to survive from the innate immune killing. Undoubtedly, the νSaα specific *lpl* cluster contributes to bacterial invasion into human keratinocytes as significantly less Δ*lpl* mutant bacteria were found intracellularly in cultured keratinocytes compared with its intact parental strain. Moreover, the described effect could be reversed by the complemented mutant (Nguyen et al., 2015). The *in vitro* effect of Lpl proteins was also confirmed by a murine skin invasion model using shaving and tape-stripping, as higher bacterial counts were found in mice that were epicutaneously infected with the USA300 parental strain as compared to the Δ*lpl* mutant (Nguyen et al., 2015). Interestingly, it was recently shown that unlipidated Lpl1 protein prompts *S. aureus* host cell invasion *via* direct interplay with the Hsp90 receptor (Tribelli et al., 2020).

In the skin invasion model, the epidermis was disrupted by tape-stripping. In contrast, subcutaneous injection of bacteria bypassed the natural protection layers such as epidermis and dermis. However, a similar phenomenon was also observed when we subcutaneously infected the mice with the SA113- or the Newman parental strain, as these *S. aureus* Lpp-expressing strains induced fulminant bacterial growth in local skin of mice independent of host TLR2 signaling in contrast to their *lgt*-deficient mutant counterparts (Mohammad et al., 2021). This was accompanied by skin abscess formation and delayed wound healing in the local tissues of mice (Mohammad et al., 2021). Furthermore, upon subcutaneous injection with a complemented mutant strain, SA113Δ*lgt* (pRB474::*lgt*), used in order to validate the impact of *S. aureus* Lpps in the skin infection model, the inoculated mice displayed more severe skin lesions and higher bacterial loads in the mouse skin homogenates in comparison to the SA113Δ*lgt* mutant strain (Mohammad et al., 2021). In addition, the levels of leukocyte chemoattractants (MIP-2, KC and MCP-1) and myeloperoxidase (MPO) in those skin homogenates were shown to be upregulated (Mohammad et al., 2021).

In the murine skin model, it was also revealed that subcutaneous injection of purified *S. aureus* Lpps in mice was associated with enhanced levels of tissue factor (TF) and plasminogen activator inhibitor-1 (PAI-1) in a lipid-moiety-dependent manner (Mohammad et al., 2021). Similarly, co-injection of purified *S. aureus* Lpp and live *S. aureus* bacteria deficient in Lpp expression also displayed upregulated expression of PAI-1, in a lipid-moiety-dependent fashion (Mohammad et al., 2021), suggesting that Lpp expression causes an imbalance of the coagulation/fibrinolysis hemostasis in the murine skin model. Importantly, in fibrinogen-depleted mice the Lpp-induced effects were fully abolished (Mohammad et al., 2021). This indicates that *S. aureus* Lpp expressing bacteria promote fibrous capsule formation upon skin infection by utilizing fibrinogen, thus shielding the bacteria from immune killing. This further shows that different *S. aureus* Lpp-expressing strains give rise to similar virulent characteristics in two different skin infection models. Purified *S. aureus* Lpps were shown to promote murine skin inflammation through activation of dendritic cells in an intradermal injection model (Saito and Quadery, 2018). Likewise, induced skin inflammation was also observed in our subcutaneous skin injection model (Mohammad et al., 2021).

#### The plausible mechanism for distinct roles of *Staphylococcus aureus* Lpps in different models

The cellular mechanism behind the lower bacterial load in the murine knee joints intra-articularly inoculated with Lpp-expressing *S. aureus* was elucidated using co-injection of live bacteria with purified *S. aureus* Lpp or synthetic lipopeptides. This resulted in bacterial eradication in the knee joints – a phenomenon that was mediated through TLR2-dependent responses with neutrophils acting as the main phagocytic cell engulfing the bacteria (Mohammad et al., 2019). In fact, enhanced levels of the neutrophil attracting chemokines, KC and MIP-2, were observed in the supernatants of knee homogenates (Mohammad et al., 2019). In the skin model, instead, co-injection of live bacteria with purified *S. aureus* Lpps resulted in opposite effects as the skin damage worsened by displaying more severe lesions and abscess frequencies, along with increased local bacterial persistence. This was associated with increased levels of the neutrophil chemoattractant chemokines, MIP-2 and KC, and the critical recruiter of monocytes/macrophages, MCP-1, as well as MPO in the skin homogenates of mice in a lipid-moiety-dependent manner (Mohammad et al., 2021). Our data indicate that not only purified *S. aureus* Lpps but also a mixture of Lpps and live bacteria are able to activate a powerful innate immune response in the model of local knee arthritis, whereas the same mixture plays a more beneficial role for the bacteria in the skin model. Elevated levels of the same chemokines were also observed in the skin homogenates upon subcutaneous injection with purified *S. aureus* Lpps exclusively, in a lipid- and a dose-dependent fashion through TLR2 (Mohammad et al., 2021), strongly suggesting that Lpps play a potent role in triggering local inflammatory responses in different organs.

In the hematogenous septic arthritis model, expression of Lpp in *S. aureus* increased mortality, weight loss and cytokine production, and decreased bacterial clearance independent of TLR2, indicating the important role of Lpp in bacterial fitness and virulence (Mohammad et al., 2020). As Lpp receptor, TLR2 plays a role in the host defense against infection, as TLR2 deficient mice infected with the Newman parental strain displayed enhanced arthritis symptoms as well as increased weight loss, mortality and bacterial burden in kidneys compared to the wild-type controls (Mohammad et al., 2020). This result was not so surprising since several reports previously showed that TLR2 deficient mice are significantly more susceptible to *S. aureus*-induced infections and display increased bacterial loads in different organs in comparison to their wild-type counterparts (Takeuchi et al., 2000; Miller et al., 2006; Sun et al., 2006; Stenzel et al., 2008; Schmaler et al., 2009).

In the local knee arthritis model, we demonstrated that the destructive arthritis caused by Lpp is TLR2-dependent (Mohammad et al., 2019), possibly due to an excessive inflammatory reaction. However, in the hematogenous septic arthritis model, we showed that the destructive arthritis caused by Lpp-expressing *S. aureus* was TLR2-independent. The multifunctionality of Lpps, i.e., in nutrition and fitness, bacterial survival and pathogenicity during host-interactions, or its ability to evade immune recognition or to trigger various immune responses upon invasion, are all of significant importance during infection with live *S. aureus* expressing Lpp.

**Figure 3:**
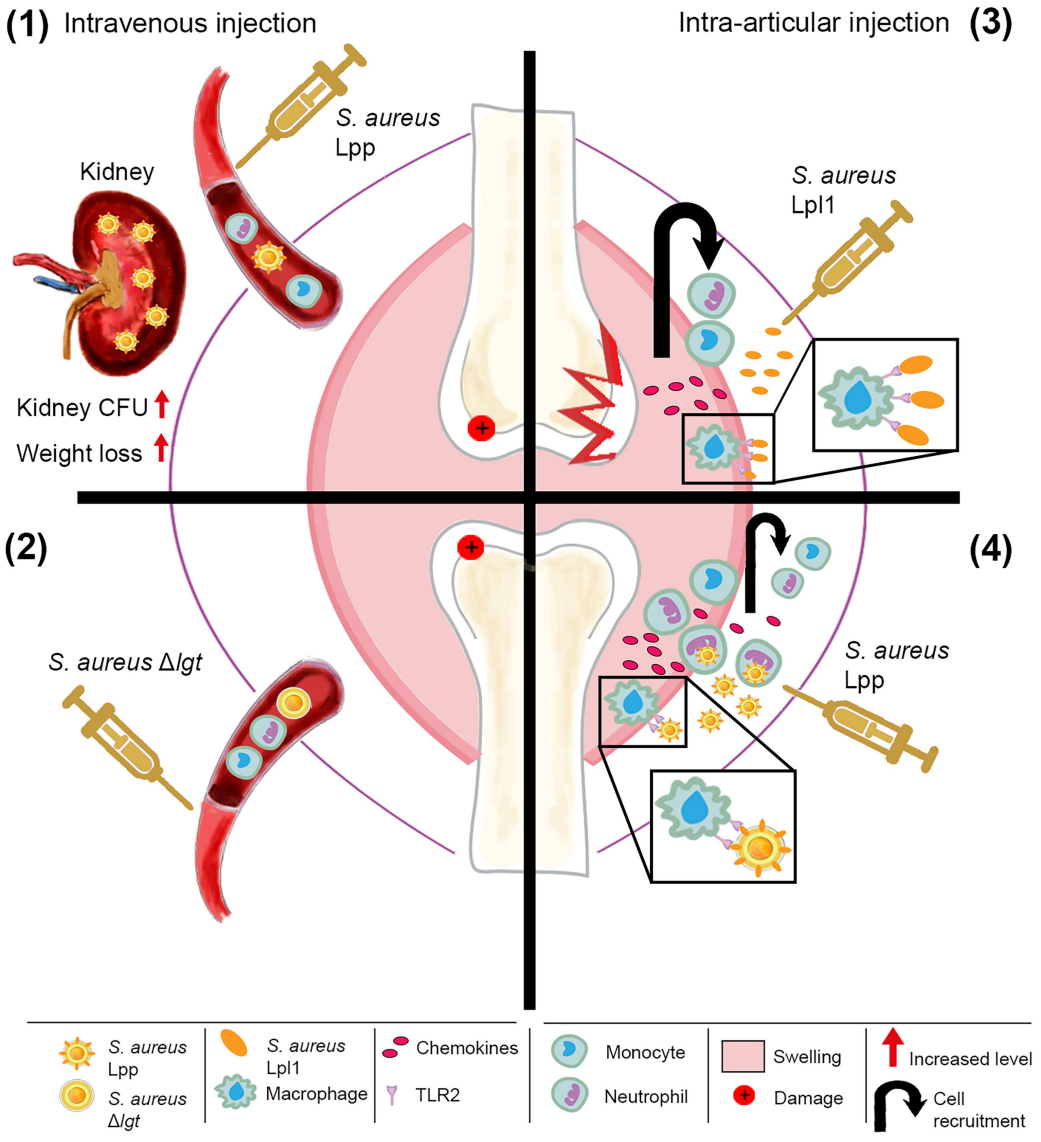
Schematic illustration of the effects of *Staphylococcus aureus* lipoprotein (Lpp) in hematogenous and local *S. aureus* arthritis models. **Left panel:** *S. aureus* parental strain, expressing Lpp (*S. aureus* Lpp), significantly aggravates systemic infection with increased mortality, weight loss, and bacterial burden in the kidneys **(1)** compared to the derivative *lgt* mutant strain, lacking Lpp (*S. aureus* Δ*lgt*) **(2)**. However, both *S. aureus* strains have similar outcomes with regard to bone erosion. **Right panel:** Lpp has dual effects in the local knee model. Intra-articular injection of purified Lpp (*S. aureus* Lpl1) induces rapid TLR2-dependent infiltration of phagocytes. Moreover, Lpl1 causes severe joint inflammation and bone erosions dependent on monocytes/macrophages through TLR2 **(3)**. In contrast, live *S. aureus* Lpps act as adjuvants, triggering recognition by TLR2 and subsequent neutrophil recruitment, leading to more efficient bacterial killing and diminished bone destruction **(4)**. CFU, colony-forming units.

A schematic illustration of the effects of *S. aureus* Lpp in hematogenous and local *S. aureus* arthritis models is shown in Figure 3.

Lpps have emerged as an important factor also during the pathogenesis of *S. aureus* systemic infections (Schmaler et al., 2009; Mohammad et al., 2020). Furthermore, another study indicated that *S. aureus* Δ*lgt* mutant was hypervirulent in contrast to its parental strain, in a murine sepsis model (Bubeck Wardenburg et al., 2006). Surprisingly, in the same article, the authors demonstrated that the *lsp*-deficient strain exhibited attenuated virulence, which is more in line with many other studies (Bubeck Wardenburg et al., 2006). One could speculate that these contradictory findings might be due to variations in animal species and age, bacterial dose or bacterial strain, duration of the course of infection, or that the generation of the *lgt* deletion mutant might have been performed differently in the reported studies, possibly underlying these divergent outcomes on lipidation and maturation of staphylococcal Lpp in murine staphylococcal sepsis. Nevertheless, from the majority of the previous studies we conclude that *S. aureus* Lpps are pathogenic in systemic and skin infections. *Staphylococcus aureus* Lpps and their differential roles in different *in vivo* models are summarized in Table 2.

## Concluding remarks

*Staphylococcus aureus* Lpps play a differential role depending on the affected organ and route of injection, as described below.

Lpps display a dual function in local *S. aureus* arthritis models. On the one hand, purified Lpp, but not toxic shock syndrome toxin-1 (TSST-1) or PGN, induced chronic macroscopic arthritis. Intra-articular injection with Lpps induced rapid TLR2-dependent infiltration of monocytes/macrophages and neutrophils. Furthermore, *S. aureus* Lpps caused severe joint inflammation and bone erosions, which were mediated by monocytes/macrophages through TLR2. On the other hand, Lpp expression in *S. aureus* led to reduced bacterial burden in the arthritic knee joints. The observed phenomenon was due to Lpp acting as adjuvant and triggering recognition by TLR2 followed by subsequent neutrophil recruitment, leading to more efficient bacterial killing and diminished bone destruction.

*Staphylococcus aureus* Lpps were found to be prominent virulence factors independent of host TLR2 expression. Mice that were intravenously inoculated with the *S. aureus* Lpp-expressing parental strain succumbed more to the disease, had increased weight loss, and exhibited impaired bacterial clearance in their kidneys, than mice inoculated with the *S. aureus* Δ*lgt* mutant strain, lacking Lpp expression. Notably, the worst outcome was observed in mice lacking TLR2 and inoculated with the *S. aureus* parental strain, strongly indicating the protective role of TLR2 in hematogenous spread of *S. aureus*-induced septic arthritis. However, in contrast to the local septic arthritis model, *S. aureus* Lpps exhibited a limited role in bone erosion.

*Staphylococcus aureus* Lpps were associated with severe inflammatory response in the skin model. The observed skin lesions and inflammation were mediated through TLR2-dependent mechanisms. Lpp contributed to a similar influx of innate immune cells as observed in the local knee arthritis model with monocytes/macrophages as well as neutrophils being recruited to the local tissue. In addition, subcutaneous injection of *S. aureus* parental strain was associated with elevated bacterial burden in the skin biopsies and more severe skin lesions. Importantly, Lpp expression initiated the activation of the coagulation and inhibition of fibrinolysis, and resulted in enhanced local fibrin deposition and abscess capsule formation in murine skin infection, whereas depletion of leukocytes and fibrinogen resulted in the total abrogation of effects induced by *S. aureus* Lpp. Such findings indicate that *S. aureus* Lpp-expressing bacteria utilize a “lockdown” strategy, consequently preventing the bacteria from being killed by the immune system, which represents a novel bacterial immune evasion mechanism.

**Figure 4:**
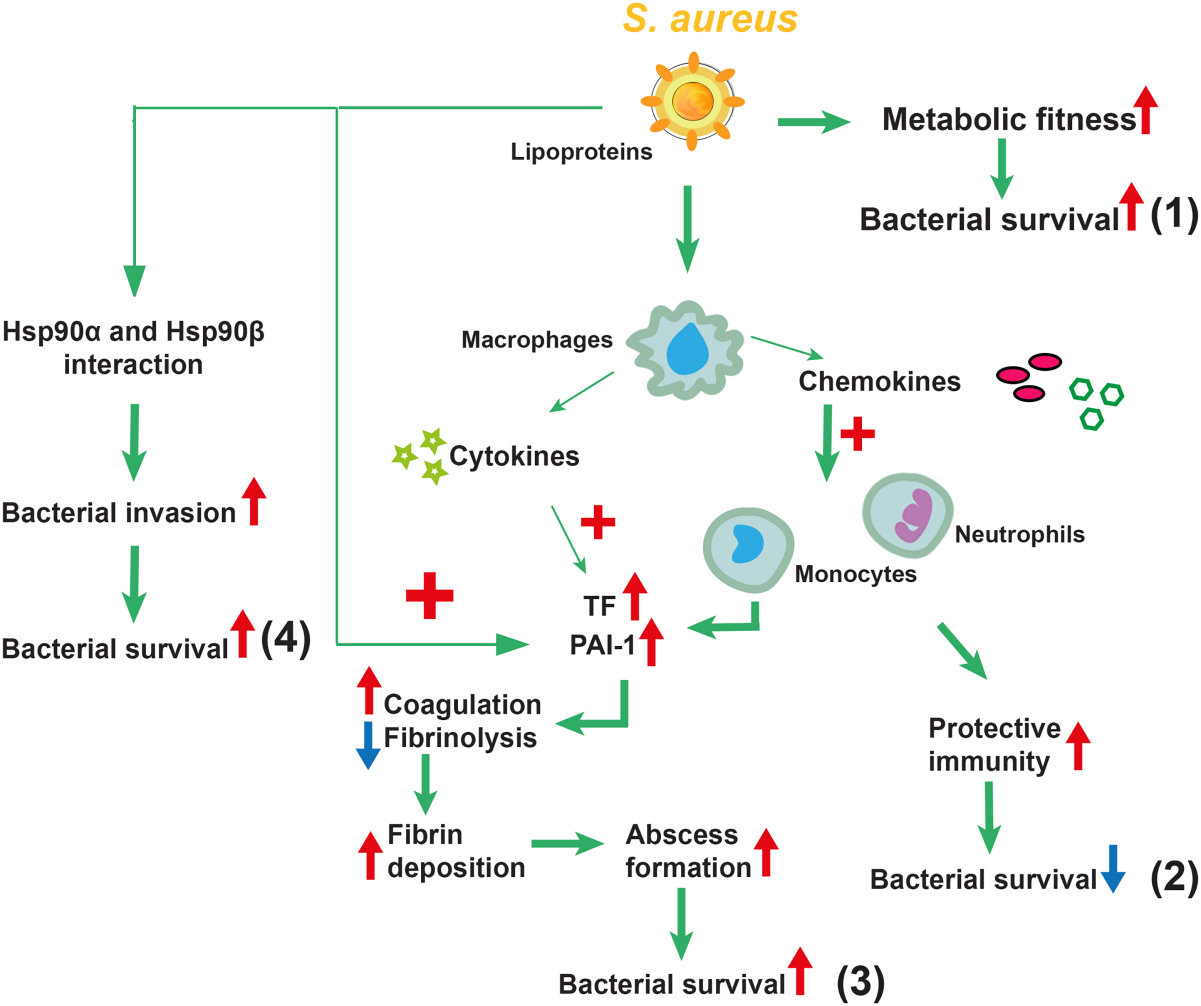
Overview of the proposed functions of lipoproteins (Lpps) in *Staphylococcus aureus* infections. *S. aureus* Lpps play a distinct role depending on the route of infection. Following systemic administration of Lpp-expressing *S. aureus*, increased metabolic fitness and increased bacterial survival are observed **(1)**. Local administration of *S. aureus* Lpps has dual effects depending on the affected organ. In the knee joint infection, Lpps have a protective role by triggering the innate immunity, leading to more efficient bacterial elimination with subsequent diminished bone destruction **(2)**. In skin infection, in contrast, Lpp-expressing *S. aureus* leads to increased abscess formation, facilitating the bacteria to evade the innate immunity and resulting in increased bacterial survival **(3)**. Furthermore, Lpp prompts *S. aureus* host cell invasion *via* direct interplay with the heat shock proteins Hsp90α and Hsp90ß, consequently leading to bacterial survival **(4)**.

Overall, Lpp maturation contributed to staphylococcal immune evasion. An overview of the proposed functions of Lpps in *S. aureus* infections is summarized in Figure 4.

## Future perspectives

As mentioned above, bacterial Lpps exist in two different forms depending on the lipid moiety, i.e., diacylated and triacylated Lpps. Our future aim is to further explore and compare the different lipid-moieties of the staphylococcal species *S. aureus* and *S. carnosus*, as well as the synthetic lipopeptides, Pam_3_CSK_4_ and Pam_2_CSK_4_, and investigate their role in the induction of bone erosion. To our knowledge, there are currently no studies available regarding the role of diacylated and triacylated Lpps on the induction of bone damage. We hypothesize that the diacylated Lpp structure is a more potent inducer of bone erosion. As outlined before, the degree of acylation of the lipid moiety impacts the immune response. Importantly, Nguyen et al. recently showed that the lipid-moieties of Lpps from different bacterial species significantly differ regarding their immune stimulatory activity (Nguyen et al., 2017). We also recently revealed that Lpps cause bone resorption in a mouse model of *S. aureus* septic arthritis, and that the diacylated lipid moiety, Pam_2_CSK_4_, was more potent in inducing bone resorption than the triacylated lipid moiety, Pam_3_CSK_4_ (Schultz et al., 2022). In addition, earlier studies conducted on skin resident cells demonstrated that di- but not triacylated Lpps suppressed the immune tolerance, a phenomenon that was mediated through IL-6 release, and the subsequent induction and accumulation of myeloid-derived suppressor cells (Skabytska et al., 2014).

Finally, it has recently been proposed that the combination of different staphylococcal MAMPs might exert an additive or possibly even a synergistic effect in immune stimulation (Nguyen and Götz, 2016). *Staphylococcus aureus* Lpps as well as PGN are known MAMPs of *S. aureus* (Nguyen and Götz, 2016). It is known that most staphylococcal infections are successfully promoted by the coordinated action of different virulence factors rather than a single virulence factor (Fournier and Philpott, 2005). In fact, co-stimulation of dendritic cells with PGN and synthetic lipopeptide enhanced immune stimulatory effects compared to PGN or lipopeptide stimulation alone (Schaffler et al., 2014). Therefore, we plan to determine whether Lpps and PGN act synergistically in staphylococcal skin infections.

As we have seen through the above studies, Lpp gave rise to different outcomes in different organs. What does this depend on? We speculate that the different clinical outcomes might be explained by the anatomic differences, composition difference of immune cells and distribution of blood vessels in the different organs. However, more detailed studies are warranted in the future to answer this question.

## Author contributions

MM: wrote the manuscript. AA, M-TN, FG, RP, and TJ: critically revised the manuscript. Some content of this article is a part of the Ph.D. thesis of MM. All authors contributed to the article and approved the submitted version.

## Funding

This work was supported by the Swedish Medical Research Council (grant number 523-2013-2750 to TJ); grants from the Swedish state under the agreement between the Swedish Government and the county councils, the ALF-agreement (grant number ALFGBG-823941 to TJ, ALFGBG-926621 to RP); E och K.G. Lennanders stipendiestiftelse to (MM and AA); Sahlgrenska University Hospital Foundations to (MM); Rune och Ulla Amlövs Stiftelse för Neurologisk, Reumatologisk och Audiologisk Forskning to (MM and AA); Inger Bendix Stiftelse för Medicinsk Forskning to (MM); Kungl. Vetenskapsakademiens stiftelser (grant number ME2015-0119 to AA); Magnus Bergvalls Stiftelse (grant numbers 2017-01958 and 2018-02797 to AA); and Institute of Medicine, Gothenburg University. M-TN acknowledges the Innovative Medizinische Forschung (IMF) Grant provided by the Medical Faculty Münster (number NG 122106). FG was supported by the Deutsche Forschungsgemeinschaft the Germany’s Excellence Strategy—EXC 2124 (Project no. 390838134) “Controlling Microbes to Fight Infections” (CMFI). We are grateful to Libera Lo Presti (Excellence Cluster 2124 “Controlling Microbes to Fight Infections” (CMFI), University of Tübingen, Germany) for critical reading of the manuscript.

## Conflict of interest

The authors declare that the research was conducted in the absence of any commercial or financial relationships that could be construed as a potential conflict of interest.

## Publisher’s note

All claims expressed in this article are solely those of the authors and do not necessarily represent those of their affiliated organizations, or those of the publisher, the editors and the reviewers. Any product that may be evaluated in this article, or claim that may be made by its manufacturer, is not guaranteed or endorsed by the publisher.

## Abbreviations

Lpps: Lipoprotein(s)
*lpl*: lipoprotein-like lipoprotein genes
Lpl1: lipoprotein-like protein 1
*lgt*: diacylglyceryl transferase enzyme encoding gene
*lsp*: signal peptidase II encoding gene
*lns*: lipoprotein *N*-acylation transferase system
*lnt*: apolipoprotein *N*-acyltransferase
LTA: lipoteichoic acid
PGN: peptidoglycan
TSST-1: Toxic shock syndrome toxin-1
EVs: extracellular vesicles
MAMP: microbe-associated molecular pattern
PRRs: pattern recognition receptors
TLR2: Toll-like receptor 2
IL: interleukin
KC: keratinocyte-derived chemokine
MCP-1: monocyte chemoattractant protein 1
MIP-2: macrophage inflammatory protein-2
MPO: myeloperoxidase
PAI-1: plasminogen activator inhibitor-1
TF: tissue factor
*S. aureus*: *Staphylococcus aureus*
